# Specificity of RSG-1.2 Peptide Binding to RRE-IIB RNA Element of HIV-1 over Rev Peptide Is Mainly Enthalpic in Origin

**DOI:** 10.1371/journal.pone.0023300

**Published:** 2011-08-10

**Authors:** Santosh Kumar, Debojit Bose, Hemant Suryawanshi, Harshana Sabharwal, Koyeli Mapa, Souvik Maiti

**Affiliations:** Proteomics and Structural Biology Unit, Institute of Genomics and Integrative Biology, CSIR, Delhi, India; National Institutes of Health, United States of America

## Abstract

Rev is an essential HIV-1 regulatory protein which binds to the Rev responsive element (RRE) present within the *env* gene of HIV-1 RNA genome. This binding facilitates the transport of the RNA to the cytoplasm, which in turn triggers the switch between viral latency and active viral replication. Essential components of this complex have been localized to a minimal arginine rich Rev peptide and stem IIB region of RRE. A synthetic peptide known as RSG-1.2 binds with high binding affinity and specificity to the RRE-IIB than the Rev peptide, however the thermodynamic basis of this specificity has not yet been addressed. The present study aims to probe the thermodynamic origin of this specificity of RSG-1.2 over Rev Peptide for RRE-IIB. The temperature dependent melting studies show that RSG-1.2 binding stabilizes the RRE structure significantly (*ΔT*_m_ = 4.3°C), in contrast to Rev binding. Interestingly the thermodynamic signatures of the binding have also been found to be different for both the peptides. At pH 7.5, RSG-1.2 binds RRE-IIB with a *K_a_* = 16.2±0.6×10^7^ M^−1^ where enthalpic change *ΔH* = −13.9±0.1 kcal/mol is the main driving force with limited unfavorable contribution from entropic change *TΔS* = −2.8±0.1 kcal/mol. A large part of *ΔH* may be due to specific stacking between U72 and Arg15. In contrast binding of Rev (*K_a_* = 3.1±0.4×10^7^ M^−1^) is driven mainly by entropy (*ΔH* = 0 kcal/mol and *TΔS* = 10.2±0.2 kcal/mol) which arises from major conformational changes in the RNA upon binding.

## Introduction

The large pre mRNA transcript produced by HIV-1, during its early phase of lifecycle, undergoes alternative splicing to generate small mRNA transcripts for production of certain regulatory proteins. Two of the key regulatory proteins essential for the replication of virus inside the host cell are Tat, and Rev [1]–[3]. Rev plays a crucial role for the export of unspliced mRNA, from nucleus to the cytoplasm, upon binding to the Rev Response Element (RRE). RRE is a 234 nucleotide long RNA present in the *env* gene [4]–[9]. This binding event has immense importance for the production of intact virions and propagation of virus inside the host [10], [11].

Different biochemical studies have shown that, Rev-RRE interaction is primarily mediated through an arginine rich motif (ARM) of the protein [12], [13] which binds to IIB region of RRE in α-helical conformation [14]. This interaction brings about conformational changes to the RNA and facilitates binding of up to 8 Rev monomers [15]–[18] in a cooperative manner. This multimeric RNA-protein complex is then transported to the cytoplasm with the help of exportin-1 and other nuclear protein complexes [19]–[22]. Biochemical studies have identified the critical bases in upper stem region of RRE-IIB essential for Rev binding [23]–[25]. In addition, important residues of Rev protein involved in Rev-RRE binding have also been identified by different *in vitro* selection, chemical modification and mutagenesis studies [18], [26]–[29]. Based on these studies many groups have tried to generate peptides with increased affinity and specificity to block the Rev-RRE-IIB complex formation. One such peptide RSG-1.2, evolved through multiple rounds of mutations and selection, is known to bind to RRE-IIB with a 7 fold higher affinity and 15 times higher specificity than Rev [30]. NMR structures of RRE-IIB in complex with Rev [31] and with RSG-1.2 [32], [33] show different binding modes for the two peptides. In case of the Rev-RRE-IIB complex, the α helical region of the peptide is inserted into the wide major groove of the RRE RNA, thereby enabling the side chains of amino acids to interact with the bases and run parallel to the phosphate backbone [31]. On the other hand, the RSG-1.2 peptide helix binds RRE in the deep groove in such a fashion that the helix axis runs perpendicular of RRE duplex [32]. NMR and crystallographic studies also show that the U72 base behaves differently in the two complexes. Rev binding causes the flipping of U72 residue towards the aqueous solution [31], whereas RSG-1.2 binding results in flipping of U72 residue inside the major groove region facilitating the stacking of U72 with Arg15 residue [32].

**Figure 1:**
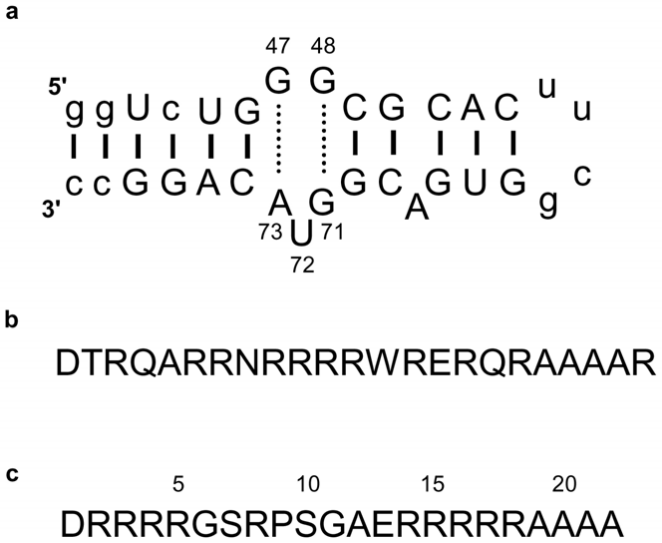
Sequence of RRE-IIB construct Rev peptide, and RSG-1.2 peptide used for studies. (a) RRE oligonucleotide representing the IIB stem loop structure of HIV-1 RRE-IIB, numbered nucleotides shows that 2-AP fluorophore, is present between A73-G47 and G71-G48 base pairs at U72 position (b) Sequence of Rev peptide (c) Sequence of RSG-1.2 peptide, indicated numbers show the position of amino acid. (non wild type nucleotides are shown in lowercase [32].)

Although it is well documented that RSG-1.2 binding to RRE-IIB is 7 fold stronger in affinity and 15 times more specific than Rev binding, the basis of this higher affinity and specificity is still unknown. A detailed thermodynamic analysis of these binding events could provide a better insight into the origin of this high affinity and specificity. We have done thermodynamic profiling of interactions of RRE-IIB (Fig. 1a) with Rev (Fig. 1b) and RSG-1.2 (Fig. 1c) using different spectroscopic and calorimetric techniques. Interestingly we found that the binding modes of two peptides are rather different. Isothermal titration calorimetry (ITC) results demonstrate binding of Rev is exclusively entropy driven, whereas RSG-1.2 binding is primarily enthalpy driven with a small entropic contribution. The difference in heat capacity change of the RNA peptide complexes also indicates the differential recognition and binding modes for the two peptides.

## Materials and Methods

### Biochemicals

The peptides, used for these studies, Rev (DTRQARRNRRRRWRERQRAAAAR) and RSG-1.2 (DRRRRGSRPSGAERRRRRAAAA) along with two mutants of RSG-1.2, whose sequences are R15D (DRRRRGSRPSGAERRDRRAAAA), R15K (DRRRRGSRPSGAERKRRRAAAA) were purchased from Hysel India Pvt. Ltd. and used without any further purification. The 32 nucleotide long stem loop sequence of RRE-IIB RNA used for these studies (GGUCUGGGCGCACUUCGGUGACGGUACAGGCC) was obtained from Ocimum Biosolutions Ltd. Netherlands. The concentration of the oligo was determined spectrophotometrically at 260 nm at 25°C using the molar extinction co-efficient 302.6×10^3^ M^−1^cm^−1^. These values were calculated by extrapolation of the tabulated values of dimer and monomer nucleotides at 25°C to high temperatures using a previously reported protocol [34].

Fluorescence labeled RNA having 2-Aminopurine (2-AP) at U72 position of RRE-IIB was obtained from Ocimum Biosolutions Ltd. Netherlands. All the stock solutions were prepared in Milli-Q water. Other reagents were commercially available and were of analytical grade quality and used without any further purification. All the experiments were performed using 10 mM sodium cacodylate buffer containing 0.1 mM EDTA and 50 mM NaCl at pH 7.5.

### CD Spectroscopy

Circular dichroism (CD) spectra were recorded on a Jasco spectropolarimeter (model J815, Japan) equipped with a thermoelectrically controlled cell holder. The cuvette used for acquiring spectra was of 1 cm in path length. CD spectra were recorded from 350 nm to 200 nm; with accumulation of 3 and averaged at 25°C. The buffer solution contained 10 mM sodium cacodylate buffer, 0.1 mM EDTA and 50 mM NaCl at pH 7.5. In a titration experiment RNA concentration was fixed at 5 µM and the peptides concentration varied from 0–25 µM.

### Temperature dependent UV Spectroscopy (UV melting)

Temperature dependent UV melting experiments were carried out using a Cary 100 (Varian) Spectrophotometer equipped with thermoelectrically controlled cell holder. A quartz cell of 1cm path length was used for all absorbance studies. Temperature dependent absorption spectra were obtained at 260 nm with rate of 0.5°C/min. increase in temperature, from 40 to 95°C. In these experiments the concentration of RNA was kept 1 µM and peptide concentration was increased from 0–3 µM. The buffer solution contained 10 mM sodium cacodylate buffer, 0.1 mM EDTA and 50 mM NaCl at pH 7.5. For each optically detected transition, the melting temperature (*T_m_*) was determined using previously described methods [35].

### Steady state fluorescence measurements

Steady state fluorescence spectra were collected using Xe 900 lamp of Edinburgh instruments Spectrofluorometer equipped with thermoelectrically controlled cell holders. The excitation of the sample was done at 320 nm wavelength and fluorescence spectra were collected from 340 nm to 450 nm. The excitation as well as emission slit width were kept at 3 nm. A quartz cell of 1 cm path length, transparent from four sides, was used. All the experiments were conducted at 25°C. RNA concentration was fixed at 0.1 µM and the peptide concentration was varied from 0–0.3 µM. The buffer solution contained 10 mM sodium cacodylate buffer, 0.1 mM EDTA and 50 mM NaCl at pH 7.5 and 25°C. The changes in the fluorescence intensity at 370 nm were monitored as a function of increase in peptide concentration. For data analysis, the observed fluorescence intensity was considered as the sum of the weighted contributions from a peptide bound and an unbound RNA form.
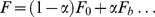
(1)


Where *F* corresponds to the observed fluorescence intensity at each titrant concentration, *F_0_* and *F_b_* are the respective fluorescence intensities of initial and final states of titration, *α* is the mole fractions of RNA in bound form. Assuming 1∶1 stoichiometry for the interaction, it can be shown that:
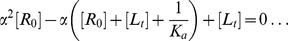
(2)


Where *K_a_* is the association constant, *[R_0_]* is the total RNA concentration and *[L_t_]* is the added peptide concentration. From Equations (1) and (2), it can be shown that:
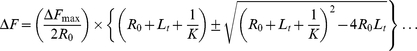
(3)


Where ΔF = F – F_0_ and ΔF_max_ = F_max_ – F_0_

The free energy of binding was calculated from the standard relation *ΔG  =  -RTlnK_a_* where *R* is the universal gas constant and *T* is the absolute temperature in Kelvin.

### Isothermal Titration Calorimetry (ITC)

Isothermal titration calorimetry measurements were conducted at 25°C on a Microcal VP-ITC (Microcal, Inc.; Northampton MA). Titration of RRE-IIB with Rev as well as RSG-1.2 were done by injecting 4 µL aliquots of 350 µM peptide from a 250 µL rotating syringe (350 rpm) into an isothermal sample chamber containing 1.5 mL of 10 µM RRE-IIB solution. Each experiment of this type was accompanied by the corresponding control experiment in which 350 µM peptide were injected into a solution of buffer alone. The duration of each injection was 8 s and the delay between the injections was 300 s, the initial delay prior to the first injection was 200 s. Each injection generated a heat burst curve (microcalories/second vs. seconds) and the area under each curve was determined by integration [using origin version 7.0 software (Microcal, Inc.; Northampton, MA)] to obtain the measure of heat associated with that injection. The buffer corrected ITC profiles for the binding of Rev and RSG-1.2 were fit with a model for two set of binding site [36]. The binding parameters that emerge from these fits are listed in the Table 1. The net enthalpy change for RRE-IIB stem loop interaction with Rev and RSG-1.2 were determined by subtraction of the heat of dilution.

**Table 1:** The binding of Rev, RSG-1.2 and RSG-1.2 mutant peptides with RRE-IIB at 25°C^a^.

Peptide	*N_1_*	*K_a1_ (M^−1^)* × 10^7^	*ΔH_1_* kcal/mol	*TΔS_1_* kcal/mol	*N_2_*	*K_a2_ (M^−1^)*× 10^5^	*ΔH_2_* kcal/mol	*TΔS_2_* kcal/mol
Rev	ND	3.1±0.4 b	0	10.2±0.2 c	ND	ND	ND	ND
RSG-1.2	1.1	16.2±0.6 b	−13.9±0.1	−2.8±0.1 c	0.9	4.5±0.7	−7.8±0.5	0.1
(R15D)	1.5	2.9±0.4	−3.9±0.1	6.2±0.3	1.5	14.2±0.2	−1.0±0.1	7.3±0.4
(R15K)	1.6	2.4±0.7	-8.7±0.1	1.3±0.2	1.6	7.0±0.2	−2.4±0.3	5.5±0.1

a The values of *N_1_, K_a1_, ΔH_1_*, *TΔS_1_*, *N_2_*, , *K_a1_*, *ΔH_2_*, and *TΔS_2_* listed here were derived from the fits of the ITC profiles shown in Fig. 6b with a model for two set of binding sites [36]. The indicated uncertainties reflect the standard deviations of the experimental data from the fitted curves (depicted as solid line in Fig. 6b).

b Values have been calculated from fluorescence binding studies and standard relationship *ΔG^b^  =  -RT lnK_a_^b^* have been used to calculate the free energy of the binding.

c Values of Δ*S* were calculated using the standard relationship Δ*G*  =  Δ*H* - *T*Δ*S*, with the indicated uncertainties reflecting the maximum possible errors in Δ*H* and Δ*G* as propagated through this equation. ND values not determined. ITC profiles of mutant peptides have been shown in Figures S3 and S4.

## Results

### RSG-1.2 induces stronger secondary structure rearrangement of RRE-IIB RNA than Rev

**Figure 2:**
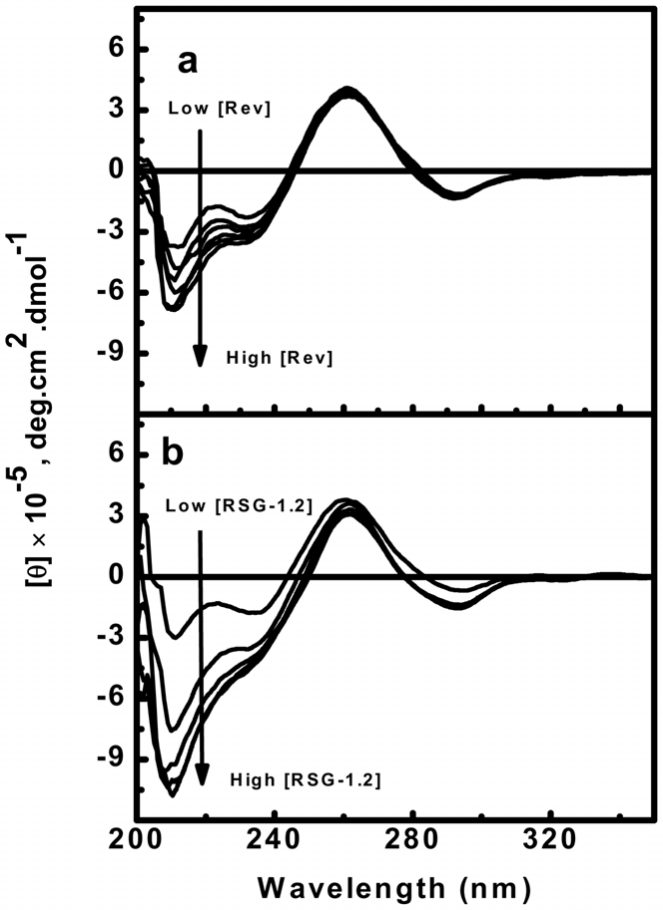
CD spectra showing the structural changes in RRE-IIB upon addition of peptides. CD spectra of RRE-IIB alone and in the presence of peptide toRRE-IIB molar ratio of 1∶1, 2∶1, 3∶1, 4∶1 and 5∶1 (a) Rev peptide (b) RSG-1.2 peptide. The concentration of RNA used is [RNA]  =  5 µM. All CD spectra were collected in buffer, consisting of 10 mM sodium cacodylate, 50 mM NaCl and 0.l mM EDTA at pH 7.5 and 25°C. Molar ellipticities, [θ], are in units of deg cm^2^ dmol^−1^, where M refers to moles of RNA strand per litre. The direction of arrow shows the increasing concentration of peptides.

Circular dichroism can be used to detect any kind of ligand induced structural perturbation in duplex upon binding. In order to detect structural changes upon binding of Rev and RSG-1.2 peptide, CD spectra of RRE-IIB were collected in the presence of different concentrations of the peptides. CD spectra were recorded using a fixed concentration of RRE-IIB at 5 µM and varying the molar ratio of peptide to RNA from 0∶1 to 5∶1. The CD spectra of RNA alone (Fig. 2) has positive maxima at 265 nm and a weak negative minima at 240 nm followed by a strong negative peak at 210 nm, characteristic of A-form RNA structure. The changes in the RRE-IIB structure brought upon addition of Rev peptide are shown in Fig. 2a. Addition of Rev peptide induces the structural changes in RRE-IIB in a concentration dependent manner. As shown in Fig. 2a, with increasing concentration of Rev peptide, the CD signal became more negative at 240 nm and 210 nm, whereas no significant change was observed at 265 nm. The structural changes in RRE-IIB, brought upon RSG-1.2 addition are shown in Fig. 2b. Similar to Rev binding, RSG-1.2 binding also shifts the CD signals towards a more negative value at both 240 nm and 210 nm wavelengths in a concentration dependent manner. However, a comparative analysis reveal that the changes in CD signal is more prominent in case of RSG-1.2 binding than that of Rev binding. CD signal at 265 nm remain unchanged in both cases. CD spectra of different RSG-1.2 mutant peptides interacting with RRE-IIB reveal that all the mutant peptides bring about approximately similar extent of secondary structure change (Fig. S1). CD spectra of only peptides (both Rev and RSG-1.2) in 25 µM concentration have also been taken to show negligible contribution of peptide to the binding spectra of RRE-IIB RNA (Fig. S2).

### RSG-1.2 stabilizes the RRE-IIB significantly in contrast to Rev

**Figure 3:**
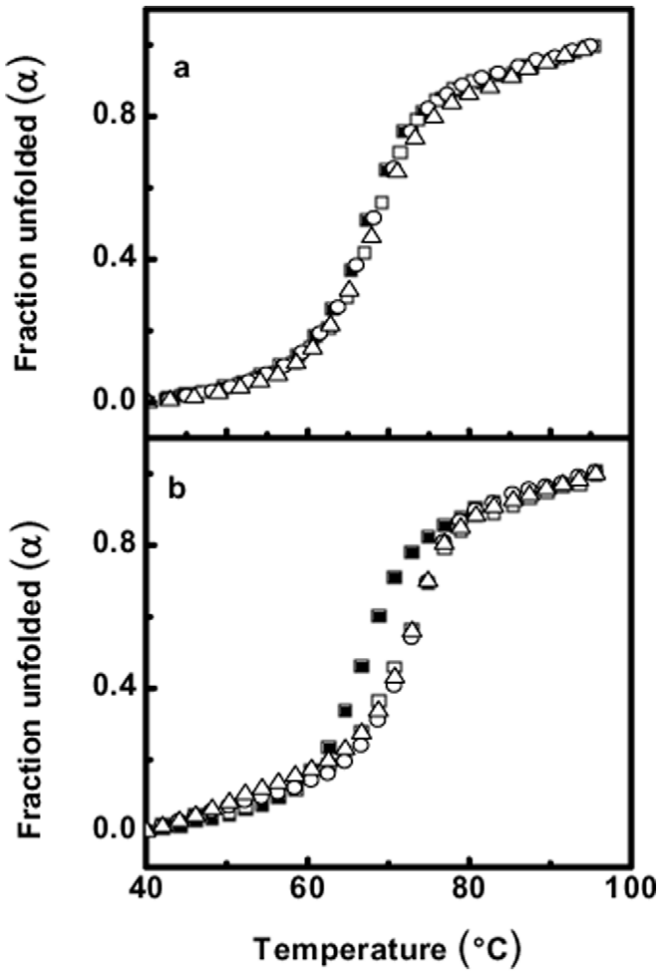
UV melting profile of RRE-IIB and the effect of peptides on its stability. UV melting profile of RRE-IIB alone and in the presence of different molar ratio of Peptide: RRE-IIB 0∶1 (!), 1∶1 (µ), 2∶1 (−), 3∶1 (8), (a) with Rev peptide (b) with RSG-1.2 peptide. All UV melting spectra were collected at 260 nm and in a buffer containing 10 mM sodium cacodylate, 50 mM NaCl and 0.l mM EDTA at pH 7.5.

Ligand induced enhancement in duplex thermal stability is an important tool to investigate the interaction between nucleic acid and their interacting partner. Thermal denaturation of RRE-IIB was carried out in the presence of different concentration of peptides (both Rev and RSG-1.2) to see the effect of their binding on the stability of RNA structure and simultaneously stoichiometry of binding was also estimated. The concentration of RNA used was 1 µM and the effect of peptide binding on the stability of RNA duplex was evaluated using different molar ratios of peptide. The molar ratio of peptide to RNA used was 0∶1, 1∶1, 2∶1 and 3∶1. In Fig. 3, unfolded fraction of RNA (*α*) has been plotted against the temperature (in °C)and as shown in Fig. 3a and 3b, the melting temperature (*T_m_*) of RRE-IIB is 67.3°C and both the peptides have differential effect on stability of RNA upon binding. In Fig 3a the melting profiles of RNA alone and in the presence of different concentrations of Rev peptide has been shown. At a peptide to RNA molar ratio of 1∶1, binding of Rev increases the *T_m_* of RNA by only 1°C, whereas at higher ratios negligible changes were observed. Similarly Fig. 3b shows the melting profile of RRE-IIB alone as well as in the presence of different concentration of RSG-1.2 peptide. As it is clearly evident, RSG-1.2 binding increases the *T_m_* of RRE-IIB from 67.3 to 71.6°C at 1∶1 molar ratio of peptide to RNA. Furthermore, at peptide to RNA molar ratio of 2∶1 and 3∶1, only negligible changes in *T_m_* were observed. Thus RSG-1.2 binding stabilizes the RRE-IIB structure by 4.3°C, which is significantly higher than the stabilization brought about by Rev binding. These results apparently suggest that Rev as well as RSG-1.2 both bind to RRE-IIB in 1∶1 stoichiometry.

### RSG-1.2 binds to RNA-IIB with 5 fold higher affinity than that of Rev

**Figure 4:**
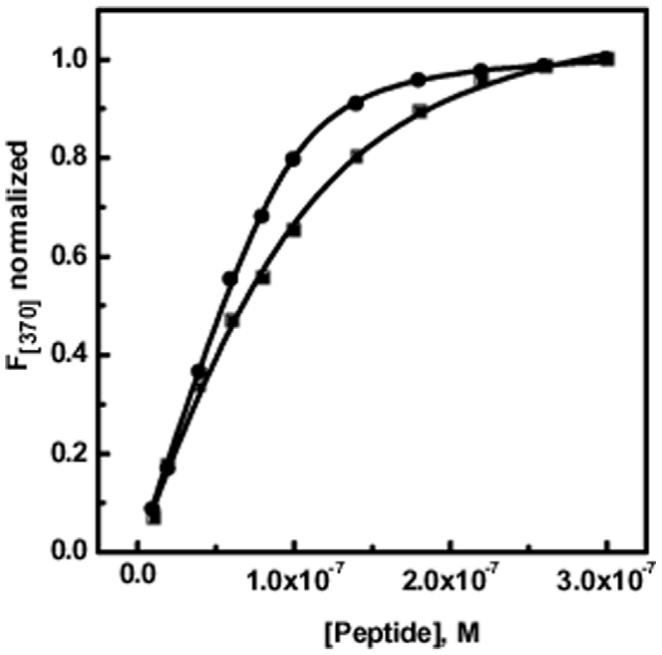
Fluorescence titrations of RRE-IIB with peptides. Fluorescence titrations of RRE-IIB (normalized at 370 nm) in the presence of increasing concentration of Rev (!) and RSG-1.2 (,) peptides. Fractional change in fluorescence intensity of 2-AP labeled RRE-IIB (at 370 nm) has been plotted against [peptide]. All titrations were performed in a buffer containing 10 mM sodium cacodylate, 50 mM NaCl and 0.l mM EDTA at pH 7.5 and 25°C. [2AP-RRE-IIB RNA]  =  0.1 µM. Solid lines represent fits of the experimental data points with equation 3. The fluorescence emission intensity at 370 nm (*F*_370_) was normalized by subtraction of the fluorescence intensity in the absence of drug (Δ*F = F -F*_0_) and subsequent division by the total calculated binding-induced change in fluorescence (Δ*F*_max_ = *F*_max_ - *F*_0_).

Fluorescence spectroscopy is a useful tool for determining binding constants of two interacting partners. We employed this tool for determining binding affinity of Rev and RSG-1.2 with the RRE-IIB RNA stem loop structure. In order to carry out fluorescence titration, RRE-IIB RNA was fluorescently labeled by incorporating 2-Aminopurine (2-AP) at position U72. There are many reports where 2-Aminopurine has been used as a probe for detecting structural changes in the RNA upon binding with small molecules [37], [38]. Melting profile of 2-AP labeled RNA shows that incorporation of 2-AP at position U72 does not have any effect on stability of RNA (data not shown). All the fluorescence titrations were performed using 10 mM sodium cacodylate buffer containing 0.1 mM EDTA and 50 mM NaCl at pH 7.5 and at 25°C. These titrations were performed at a fixed RNA concentration of 0.1 µM and the peptide was added in increasing concentration until saturation was attained. Normalized change in fluorescence intensity at 370 nm was plotted against molar concentration of peptide. In case of Rev peptide binding to RRE-IIB (Fig. 4, filled square), fluorescence intensity increased upon addition of peptide and saturation observed after 0.1 µM peptide concentration. Similarly, RSG-1.2 binding (Fig. 4, filled circle) also increased the fluorescence intensity of RRE-IIB, and saturated at ≈0.3 µM peptide concentration. After fitting the titration curves, to the equation 3, we derived peptide RNA association constant *K_a_* at 25°C temperature. The solid lines in Fig. 4 represent the best fits with correlation factor of 0.99, an observation which supports the validity of assumption of 1∶1 stoichiometry in equation 3. Fitting of Rev binding isotherm with equation 3 gave an association constant *K_a_* = 3.1±0.4×10^7^ M^−1^. On the other hand fitting of RSG-1.2 binding isotherm with the same equation gives *K_a_* = 16.2 ±0.6×10^7^ M^−1^.

### RSG-1.2-RRE-IIB complex formation is majorly enthalpy-driven while Rev-RRE-IIB is entropy-driven

**Figure 5:**
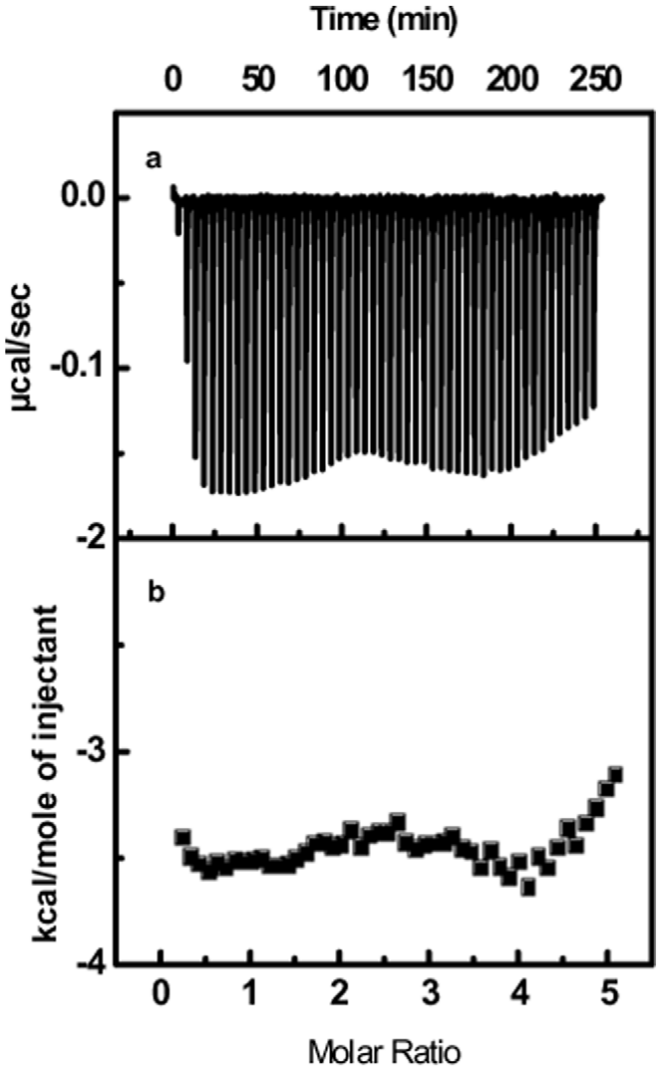
ITC titration profile of RRE-IIB with Rev. (a) the baseline corrected experimental data for Rev (b) molar heats of binding (!) plotted against the peptide to RNA molar ratio. Buffer condition was as described in the caption to Fig. 3.

**Figure 6:**
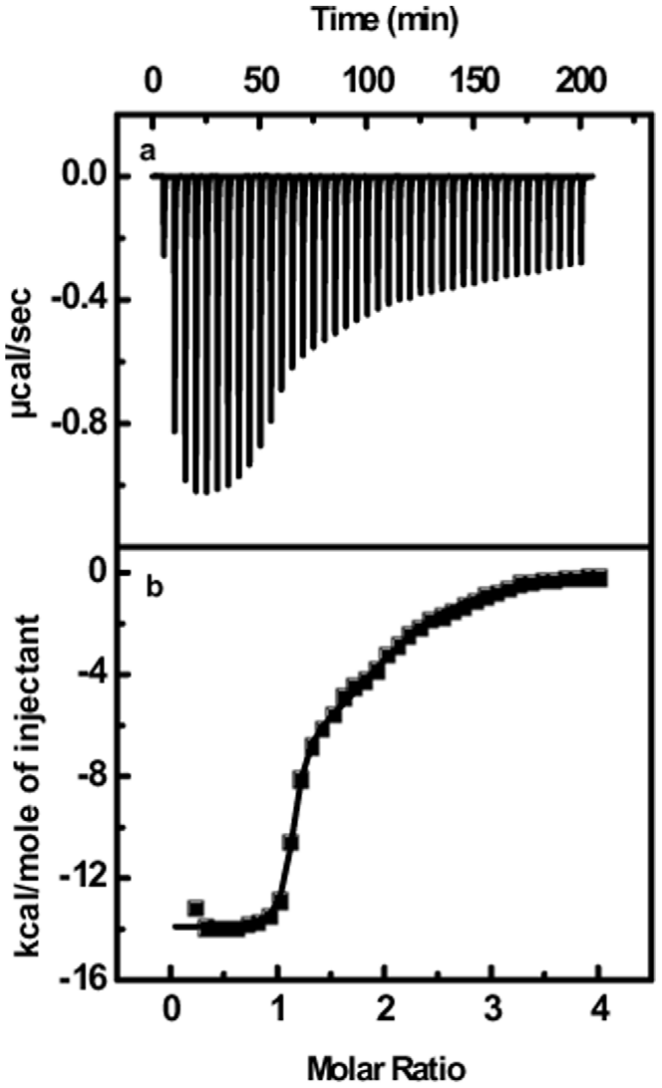
ITC titration profile of RRE-IIB with RSG-1.2. (a) The baseline corrected experimental data for RSG-1.2 (b) molar heats of binding (!) plotted against the peptide to RNA molar ratio. Buffer condition was as described in the caption to Fig. 3. Molar heat of binding is calculated by integration of the area under the curve of each heat burst using the origin version 7.0 software (Microcal, Inc.; Northampton, MA). Fitting of ITC data (shown as solid line) was done using model for two set of binding [36] given in origin version 7.0 software (Microcal, Inc.; Northampton, MA).

Isothermal Titration Calorimetry (ITC) is most widely used experimental tools to calculate different thermodynamic parameters of two binding partners. In order to characterize the binding of both the peptides with RRE-IIB RNA thermodynamically, we performed ITC experiments. Figs. 5a and 6a show the heat burst generated after each injection of 350 mM Rev and RSG-1.2 peptide respectively to a 10 µM solution of RRE-IIB RNA duplex. Each of heat burst in the panel corresponds to single peptide injection. The area under each heat burst curve was determined by integration which gives the heat associated with each injection (µcal/second). The values of heat associated with each injection were corrected for the corresponding heat of dilution derived from the peptide addition to the buffer alone under similar conditions. Figs. 5b and 6b show the corrected values of heat of each injection plotted against peptide to duplex molar ratio. The heat changes associated with Rev binding (Fig. 5a), to RRE-IIB were small, whereas heat changes associated with RSG-1.2 binding (Fig. 6a) was quite large. RSG-1.2 binding isotherm show that initial injections have large heat bursts while after saturation each heat burst represents the heat corresponding to the heat of dilution only. Solid line in Fig. 6b shows the best fit of data point of RSG-1.2 binding isotherm with a model for two set of sites [36]. RSG-1.2 binding has more negative enthalpy *ΔH*  =  −13.9±0.1 kcal/mol as compared to that of Rev binding, which has *ΔH* ≈ 0 kcal/mol (shown in Fig. 6b). The low affinity binding site with a smaller enthalpy change, in case of RSG-1.2 binding, could be due to the non-specific binding. Entropy changes associated with these binding events can be calculated by using *ΔG* values obtained from the equation *ΔG  =  -RTlnK_a_* and *ΔH* obtained from above results in equation *ΔG = ΔH – TΔS*. The thermodynamic parameters along with stoichiometry of binding of Rev and RSG-1.2 to RRE-IIB RNA have been summarized in Table 1. Gibb's free energy calculations show that RSG-1.2 binding (*ΔG* = −11.1±0.1 kcal/mol) is more favorable compared to Rev binding (*ΔG* = −10.2±0.1 kcal/mol). Analysis of Table 1 shows that RSG-1.2 binding is mainly enthalpically driven with a small negative entropic contribution (*TΔS* = −2.8±0.1 kcal/mol), on the other hand Rev binding is essentially entropically driven (*TΔS* = 10.2±0.2 kcal/mol).

Furthermore, as it is well established that residue U72 is the most labile nucleotide in RRE-IIB RNA which gets stabilized after binding with RSG-1.2 by the stacking interaction with Arg15 of the peptide. To investigate the contribution of U72-Arg15 interaction in determining the specificity of RRE-IIB-RSG-1.2 interaction, we have designed two mutants of RSG-1.2 peptide by substitution of Arg15 with Asp and Lys respectively. This substitution lowers the affinity of RSG1.2 peptide to the value similar to the Rev peptide. The thermodynamic parameters along with stoichiometry of binding of RSG-1.2 mutants to RRE-IIB RNA have been summarized in Table 1. These results with RSG-1.2 mutants confirm the major contribution of U72-Arg15 interaction towards the greater affinity of RSG-1.2 over Rev peptide. Substitution of Arg residue with a negatively charged amino acid, Asp, results in large reduction in the enthalpy change (*Δ*H = 10.0 kcal/mol). On the other hand, incorporation of similar charge bearing amino acid (Lys) results in 5.2 kcal/mol decrease in enthalpy change. These results clearly suggest that U72 –Arg15 interaction plays a crucial role in governing the selectivity and specificity of RRE-IIB-RSG-1.2 complex formation.

### RSG-1.2 binding is associated with a negative change in heat capacity while Rev binding is associated with essentially no change in heat capacity

Heat capacity changes (*ΔC_p_*), that accompany the peptide binding to RNA can be determined by performing temperature dependent ITC experiments under same buffer conditions. The standard relationship used for calculation of heat capacity changes is:
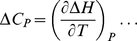
(4)


**Figure 7:**
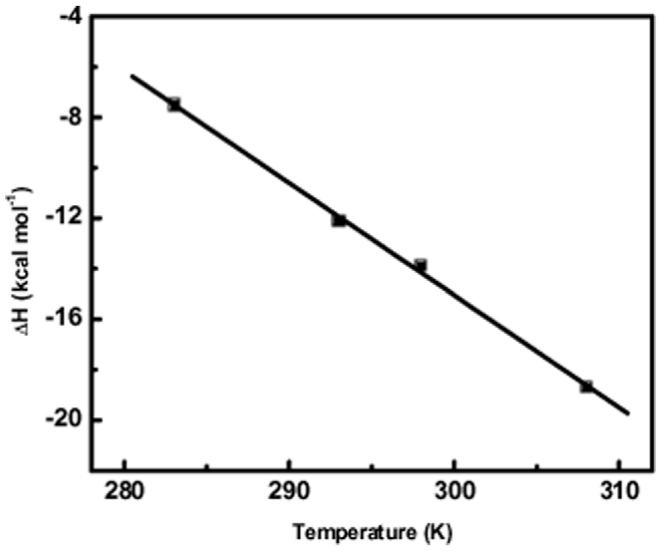
Temperature dependence of the enthalpies of binding of RSG-1.2 with RRE-IIB. Data points were fit by the linear fit analysis, with the resulting fits shown as solid line. Slope of this fitted line give the change in heat capacity (*ΔC*_p_) of the binding. Buffer condition was as described in the caption to Fig. 3.

In order to calculate the heat capacity change for the binding of peptides to RRE-IIB, we performed ITC experiment at 10°C, 20°C and 35°C, in addition to that of 25°C, under identical buffer conditions as described previously. Enthalpy change (*ΔH*) of binding at different temperatures can be plotted against the temperature and the slope of this plot gives the heat capacity change (*ΔC_p_*). In Fig. 7, the value of enthalpy change of RSG-1.2 binding to RRE-IIB has been plotted at different temperatures. Solid line represents the linear regression fit of the data points. A closer observation of Fig. 7 reveals that enthalpy (*ΔH*) of binding of RSG-1.2 to RRE-IIB becomes more negative with increasing temperature. On the other hand Rev binding to RRE-IIB does not show any change in enthalpy of binding. Thus the slope of the plot, enthalpy (*ΔH*) vs. temperature (*T*), gives heat capacity change, *ΔC_p_*  =  -444.6±15 cal mol^−1^ K^−1^, for the binding of RSG-1.2 with RRE-IIB. On the other hand Rev binding to RRE-IIB is associated with essentially no change in heat capacity. In case of ligand-nucleic acid and ligand-protein interactions it has been observed that *ΔC_p_* varies from values of -100 to -550 cal mol^−1^ K^−1^ [39]-[42]. RSG-1.2 binding to RRE-IIB is associated with a negative heat capacity change that falls within this range.

## Discussion

Gene expression in HIV-1 can be regulated at both transcriptional and post transcriptional levels by targeting different RNA protein interactions. Recent literature indicates different proteins that interact with regulatory RNA elements [43], [44]. Tat is one of the most important protein that binds to TAR RNA and regulates the transcription of mRNA. The thermodynamics of the formation of this complex has recently been revealed [37] which shows enthalpy driven binding of arginine rich motif of Tat protein to the TAR RNA. Similarly Rev also mediates the export of unspliced mRNA from nucleus to the cytoplasm by binding through its arginine rich motif to the RRE RNA. RSG-1.2 which is known to bind to RRE RNA with a higher affinity and specificity than Rev peptide could be used as a good inhibitor for RRE-Rev complex formation and hence can be of therapeutic importance. Here we performed a detailed comparative study of RRE-IIB binding with Rev and RSG-1.2 using different spectroscopic and calorimetric methods. Insight into the thermodynamic basis of these interactions can open a new avenue for developing new therapeutic molecules targeting such complexes. Thermal melting study indicates much higher stabilization of RRE-IIB structure upon binding to RSG-1.2 (*T_m_* shift 4.3°C) than Rev binding (∼1°C *T_m_* shift), albeit in both cases, the binding stoichiometry is 1∶1. Although increase in *T_m_* cannot be correlated directly with higher affinity of RSG-1.2 but this certainly indicates higher stabilizing effect of former over Rev peptide on RRE-IIB. The marginal increases in the *T_m_* at higher peptide to RNA molar ratio, above 1∶1 in both the cases, could be due to non-specific interaction with the secondary structure of RNA. More than five times increase in the association constant of RSG-1.2 – RRE-IIB complex corroborates well with the higher stabilization observed in melting study. Interestingly, ITC results not only reflect difference in binding affinity but also indicate different binding modes. As shown in Fig. 5 and 6, Rev binding with RRE-IIB has almost zero enthalpy of interaction whereas RSG-1.2 has a large negative *ΔH* value. A close inspection of different thermodynamic parameters summarized in Table 1 reveals that binding of RSG-1.2 peptide to RNA is primarily enthalpy driven. In contrast Rev binding to RRE-IIB is exclusively entropy driven. In fact, the enhanced binding of RSG-1.2 occurs despite small negative unfavorable entropic contribution to binding which is overcompensated by highly favorable enthalpic term.

The enthalpy change associated with any binding is composed of two opposite contributions. The formation of hydrogen bonds and van der Waals interactions between ligand and the host molecule generally contribute towards favorable enthalpy change, however desolvation of polar groups which occasionally happen during binding process contribute towards the unfavorable enthalpy change [45]. The large enthalpy change in case of RSG-1.2 binding to RRE-IIB indicates that RSG-1.2 establishes good number of hydrogen bonds and van der Waals interactions. Furthermore these interactions are strong enough to compensate for unfavorable enthalpy associated with desolvation. Recent molecular dynamics simulation studies have shown that Rev makes a total of 26 hydrogen bonds and RSG-1.2 makes a total of 20 hydrogen bonds and a network of continuous salt bridges spanning the major groove [46]. Though the number of hydrogen bonds involved in Rev RRE-IIB complex formation is more than RSG-1.2-RRE-IIB complex, later one has a large negative enthalpy of binding (*ΔH = *−13.9 kcal/mol) as compared to the negligible enthalpy of former one. This negligible enthalpy of Rev binding could be partially explained by different structural changes that occur in the microenvironment of the complex. It is possible that the energy released due to the formation of hydrogen bonds and van der Waals interactions is negatively compensated by the energy required for the destacking of U72 residue. On the contrary in case of RSG-1.2-RRE-IIB complex formation, the energy released due to the hydrogen bond formation and van der Waals interactions is corroborated by the energy released due to the stacking between U72 and Arg15 residue inside the major groove region. Besides, the low enthalpy change in Rev-RRE-IIB complex formation could be due to desolvation and deionization of polar groups, which negatively compensates for the energy released due to hydrogen bond formation. As reported from molecular dynamics simulation studies [46] in case of Rev-RRE-IIB binding, water molecules involved in complex formation exhibit bulk water properties and screen the charge on the peptide and RNA backbone resulting in small enthalpic contribution for complex formation. On the other hand in case of RSG-1.2 bound water molecules make direct hydrogen bonds with peptide and with RNA bases which increases the enthalpic gain by forming a low energy RNA-water-peptide complex. Contrarily it also contributes to the small unfavorable negative entropic change. Analysis of *TΔS* values of binding given in Table 1 reveal that Rev-RRE-IIB binding is mainly entropy driven with *TΔS* = 10.2±0.2 Kcal/mol, while RSG-1.2 binding has a small negative entropic contribution toward binding *TΔS* = -2.8±0.1 Kcal/mol. The large favorable entropy change in case of Rev binding to RRE-IIB may have its origin either from structural changes, conformationally labile base, desolvation or the number of water molecules released after binding. Earlier report shows that Rev binding as an α helix widens the major groove of RRE-IIB by more than 5 Å [31], whereas RSG-1.2 binding causes a minor change in the RNA backbone, as compared to Rev. Along with these reports our CD results also shows that RSG-1.2 binding leads to more structuring of RRE-IIB as compared to Rev binding. This differential structuring of RRE-IIB by both of these peptides may also contribute entropically in different ways to the binding events. In case of Rev binding, there is less structuring of RRE-IIB which leads to a more favorable entropic contribution as compared to binding of RSG-1.2. Binding of Rev also results in flipping of U72 residue towards solution side thereby increasing the flexibility of this base. On the other hand RSG-1.2 binding stabilizes the U72 residue by stacking interaction in major groove region. Taken together, all the findings suggest that in case of Rev binding all these factors contribute to the favorable entropy.

In addition ITC results of RSG-1.2 mutants interaction with RRE-IIB also support the above suppositions. When we introduce a negative charge bearing Asp residue in place of Arg15, it reduces the enthalpic contribution by more than 78%. This large reduction in enthalpic contribution could be a result of loss of U72 and Arg15 interaction corroborated with negative interaction of Asp with U72. It is also possible that insertion of Asp residue destabilizes other interactions too. But when we substitute Arg15 with similar charge bearing lysine residue the enthalpy change is larger than Asp mutant. The overall decrease in enthalpy is ≈38%, but the change in entropy is favorable as compared to that of RSG-1.2 peptide binding. For both of these mutant peptides binding affinity is also similar to the Rev peptide. These mutant peptide results show that U72 and Arg15 interaction has a major contribution towards higher affinity of RSG-1.2 and there is a large enthalpic-entropic compensation in determining the higher affinity and specificity of the RSG-1.2 peptide.

Heat capacity changes that accompany the peptide binding to RNA provide further insight into the thermodynamic mode of binding. Results obtained from temperature dependent ITC experiments shows no change in binding enthalpy for Rev peptide but in case of RSG-1.2 binding, the reaction becomes more exothermic with increasing temperature. This large negative change in heat capacity (*ΔC_p_* = -444.6±15 cal/mol*K) could arise from many factors. The different contributors to the heat capacity change are the change in solvent accessible surface area [39], [41], [47], [48], conformational changes [49]–[52] and to some extent electrostatic interactions [49], [53]. Out of these, change in solvent accessible surface, could be the major contributing factor. A negative *ΔC_p_* indicates burial of non-polar surfaces whereas burial of polar surfaces causes a positive change in *ΔC_p_*. In case of Rev binding a stretch of polar arginine residues Arg6 and Arg8-Arg11 makes hydrogen bonds or electrostatic interactions with the phosphate backbone of RNA. This results in the burial of polar groups of both peptide and RNA, and eventually led to the reduced solvent accessibility to the complex [31]. This reduction in solvent accessible surface would be expected to cause *ΔC_p_* value to be more positive. Absence of any determinable change could be a result negative compensation from the conformational changes accompanying the binding event. On the other hand large negative *ΔC_p_* value for RSG-1.2 binding to RRE-IIB could come from reduction in solvent accessible non polar surface and the conformational changes that occur upon peptide binding. As shown with NMR results, the position where polar residues bind to the phosphate backbone in case of Rev peptide is replaced by a stretch of non-polar residues. This provides two specific hydrophobic interactions Pro9 and Ala 12 with the hydrophobic surface of RNA. In addition to these specific interactions A68 of RNA also packs against the Ala rich C-terminus of RSG-1.2 peptide. All these hydrophobic interactions lead to the burial of non-polar surfaces, which in turn reduces the solvent accessible non polar surface area giving rise to large negative *ΔC_p_* for RSG-1.2 binding to RRE-IIB RNA.

### Conclusion

In conclusion, we show that selectivity of binding of RSG-1.2 over Rev peptide to RRE-IIB is mainly enthalpic in origin. The major contribution comes from the stacking between U72 of RRE-IIB and Arg15 residue of RSG-1.2 that occurs inside the major groove of the RRE-IIB which is corroborated with the energy released due to the hydrogen bond formation and van der Waals interactions. On the other hand in case of Rev binding to RRE-IIB, energy released due to the formation of hydrogen bonds and van der Waals interactions may be negatively compensated by the energy required for the destacking of U72 residue that is coupled to complex formation. These data clearly indicate that the side chain of Arg15 residue plays a major role in stabilizing the U72 base inside the RNA deep groove via stacking interactions which in turns provide higher specificity over Rev towards RRE-IIB binding.

## Supporting Information

Figure S1 **CD spectra of RRE-IIB alone and in the presence of peptide toRRE-IIB molar ratio of 1∶1, 2∶1, 3∶1, 4∶1 and 5∶1 of RSG-1.2 mutant peptides.** (a) RSG-1.2 R15D mutant peptide. (b) RSG-1.2 R15K mutant peptide. All CD spectra were collected in buffer, consisting of 10 mM sodium cacodylate, 50 mM NaCl and 0.l mM EDTA at pH 7.5 and 25°C. Molar ellipticities, [θ], are in units of deg cm^2^ dmol^−1^, where M refers to moles of RNA strand per litre. [RNA]  =  5 µM.
(TIF)

Figure S2 **CD spectra of only peptide in 25 µM concentration.** (a) Rev peptide (b) RSG-1.2 peptide, in a buffer, consisting of 10 mM sodium cacodylate, 50 mM NaCl and 0.l mM EDTA at pH 7.5 and 25°C.
(TIF)

Figure S3 **ITC profile of RSG1.2 with R15D modification with RRE-IIB at 25°C.** These titrations were performed in buffer, consisting of 10 mM sodium cacodylate, 50 mM NaCl and 0.l mM EDTA at pH 7.5.
(TIF)

Figure S4 **ITC profile of RSG1.2 with R15K modification with RRE-IIB at 25°C.** These titrations were performed in buffer, consisting of 10 mM sodium cacodylate, 50 mM NaCl and 0.l mM EDTA at pH 7.5.
(TIF)
