# Supplementary figures and images for: Specificity of RSG-1.2 Peptide Binding to RRE-IIB RNA Element of HIV-1 over Rev Peptide Is Mainly Enthalpic in Origin
Source: PLoS One. 2011 Aug 10;6(8):e23300. doi: 10.1371/journal.pone.0023300 (PMC3154333; doi:10.1371/journal.pone.0023300)

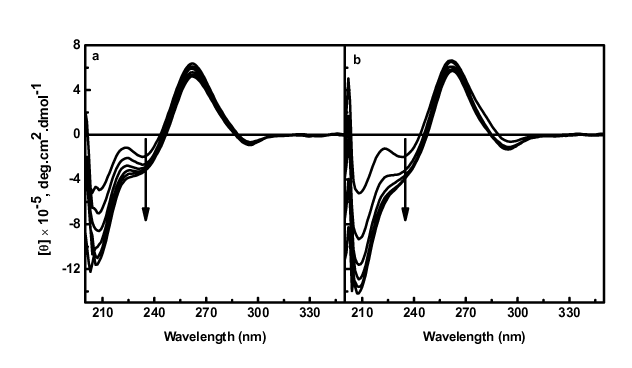

Supplement: Figure S1 — CD spectra of RRE-IIB alone and in the presence of peptide toRRE-IIB molar ratio of 1∶1, 2∶1, 3∶1, 4∶1 and 5∶1 of RSG-1.2 mutant peptides. (a) RSG-1.2 R15D mutant peptide. (b) RSG-1.2 R15K mutant peptide. All CD spectra were collected in buffer, consisting of 10 mM sodium cacodylate, 50 mM NaCl and 0.l mM EDTA at pH 7.5 and 25°C. Molar ellipticities, [θ], are in units of deg cm2 dmol−1, where M refers to moles of RNA strand per litre. [RNA] = 5 µM. (TIF) [file pone.0023300.s001.tif]

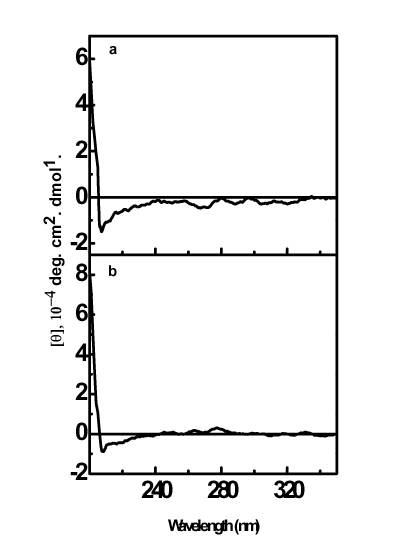

Supplement: Figure S2 — CD spectra of only peptide in 25 µM concentration. (a) Rev peptide (b) RSG-1.2 peptide, in a buffer, consisting of 10 mM sodium cacodylate, 50 mM NaCl and 0.l mM EDTA at pH 7.5 and 25°C. (TIF) [file pone.0023300.s002.tif]

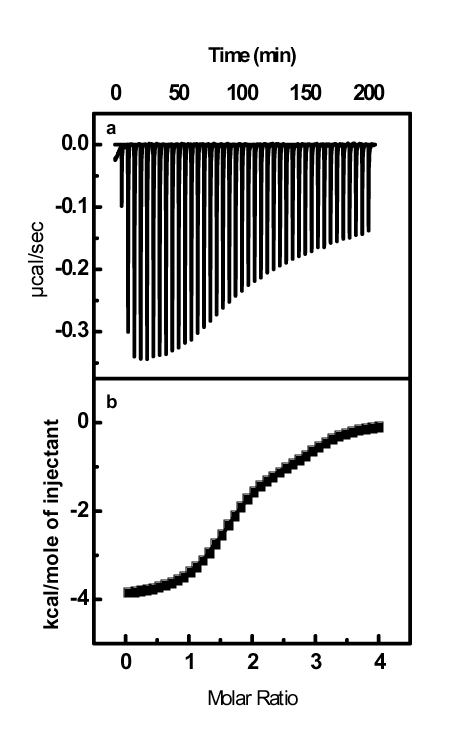

Supplement: Figure S3 — ITC profile of RSG1.2 with R15D modification with RRE-IIB at 25°C. These titrations were performed in buffer, consisting of 10 mM sodium cacodylate, 50 mM NaCl and 0.l mM EDTA at pH 7.5. (TIF) [file pone.0023300.s003.tif]

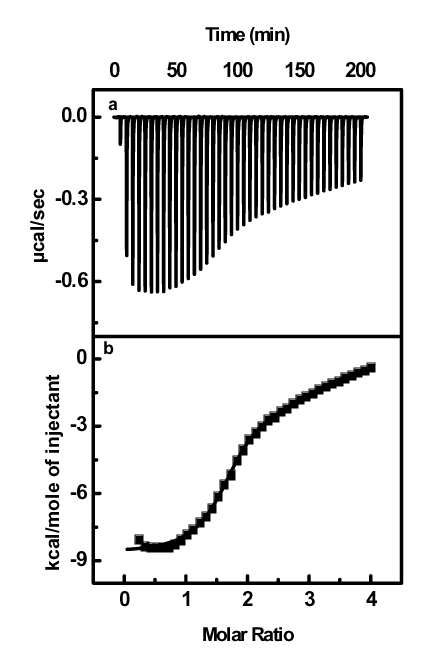

Supplement: Figure S4 — ITC profile of RSG1.2 with R15K modification with RRE-IIB at 25°C. These titrations were performed in buffer, consisting of 10 mM sodium cacodylate, 50 mM NaCl and 0.l mM EDTA at pH 7.5. (TIF) [file pone.0023300.s004.tif]
